# Notch Target Gene E(spl)mδ Is a Mediator of Methylmercury-Induced Myotoxicity in *Drosophila*

**DOI:** 10.3389/fgene.2017.00233

**Published:** 2018-01-15

**Authors:** Lisa M. Prince, Matthew D. Rand

**Affiliations:** Department of Environmental Medicine, School of Medicine and Dentistry, University of Rochester, Rochester, NY, United States

**Keywords:** *Drosophila*, notch, methylmercury, MeHg, muscle, enhancer of split, mdelta, myotoxicity

## Abstract

Methylmercury (MeHg) is a ubiquitous environmental contaminant and neurotoxicant that has long been known to cause a variety of motor deficits. These motor deficits have primarily been attributed to MeHg targeting of developing neurons and induction of oxidative stress and calcium dysregulation. Few studies have looked at how MeHg may be affecting fundamental signaling mechanisms in development, particularly in developing muscle. Studies in *Drosophila* recently revealed that MeHg perturbs embryonic muscle formation and upregulates Notch target genes, reflected predominantly by expression of the downstream transcriptional repressor Enhancer of Split mdelta [E(spl)mδ]. An E(spl)mδ reporter gene shows expression primarily in the myogenic domain, and both MeHg exposure and genetic upregulation of E(spl)mδ can disrupt embryonic muscle development. Here, we tested the hypothesis that developing muscle is targeted by MeHg via upregulation of E(spl)mδ using genetic modulation of E(spl)mδ expression in combination with MeHg exposure in developing flies. Developmental MeHg exposure causes a decreased rate of eclosion that parallels gross disruption of indirect flight muscle (IFM) development. An increase in E(spl) expression across the pupal stages, with preferential E(spl)mδ upregulation occurring at early (p5) stages, is also observed. E(spl)mδ overexpression in myogenic lineages under the Mef2 promoter was seen to phenocopy eclosion and IFM effects of developmental MeHg exposure; whereas reduced expression of E(spl)mδ shows rescue of eclosion and IFM morphology effects of MeHg exposure. No effects were seen on eclosion with E(spl)mδ overexpression in neural and gut tissues. Our data indicate that muscle development is a target for MeHg and that E(spl)mδ is a muscle-specific mediator of this myotoxicity. This research advances our knowledge of the target pathways that mediate susceptibility to MeHg toxicity, as well as a potential muscle development-specific role for E(spl)mδ.

## Introduction

Methylmercury (MeHg) is one of the most toxic forms of mercury, which has been studied extensively for its properties as a developmental neurotoxicant (Clarkson et al., 2007). Among the wide range of neurological deficits that MeHg causes, several involve motor deficits which resemble cerebral palsy, including ataxia, muscle weakness, rigidity, abnormal muscle tone and reflexes, and involuntary movements (McKeown-Eyssen et al., 1983; Harada, 1995; Roegge and Schantz, 2006). These motor deficits have primarily been attributed to MeHg targeting of neurons (Sager et al., 1982, 1984; Eto et al., 2010; Patel and Reynolds, 2013). However, few studies have examined whether MeHg targeting of the skeletal muscle system could also contribute to these motor deficits, particularly in a developmental context.

Studies in adult rats and zebrafish, however, have shown that MeHg can decrease muscle fiber size and dysregulate muscle mitochondrial shape and inhibit mitochondrial enzymes (Usuki et al., 1998; de Oliveira Ribeiro et al., 2008; Cambier et al., 2009). More recent studies in *Drosophila* have associated MeHg susceptibility to genes in core muscle developmental pathways and have suggested that developing muscle may also be a target of MeHg toxicity, through the modulation of muscle-specific signaling pathways, including the Notch pathway (Engel et al., 2012; Engel and Rand, 2014a; Montgomery et al., 2014). Notch signaling is a conserved developmental program, which is involved in cell fate decisions (Beatus and Lendahl, 1998; Artavanis-Tsakonas et al., 1999; Udolph, 2012), cellular differentiation (Shawber et al., 1996; Kuroda et al., 1999), as well as cellular fusion (Gildor et al., 2012; Bao, 2014). When activated in muscle lineages, Notch signaling inhibits differentiation, and maintains progenitors and satellite cells by promoting quiescence and self-renewal (Vasyutina et al., 2007; Mourikis et al., 2012; Wen et al., 2012).

In *Drosophila*, the adult muscle progenitors (AMPs) respond to Notch signaling in a timing- dependent manner. Notch signaling is active in quiescent AMPs in embryonic stages, but promotes proliferation in during larval stages (Aradhya et al., 2015). During indirect flight muscle (IFM) development, the Notch receptor is expressed on membranes of both the developing muscle and myoblasts, but is only active in myoblasts and not in the fibers (Bernard et al., 2006). Notch signaling regulates the expression of Twist (Anant et al., 1998), a transcription factor expressed in myoblasts, and promotes their proliferation (Gunage et al., 2014). Notch signaling is thought to maintain the undifferentiated state and myoblast pool until myoblasts receive cues to differentiate, at which point Notch signaling is downregulated (Anant et al., 1998). Both an early knockdown of Notch and a sustained activation of Notch were shown to affect IFM development (Anant et al., 1998). Notch signaling also regulates molecules involved in myoblast fusion and adhesion in *Drosophila* (Gildor et al., 2012; Bao, 2014), and therefore is important in the process of myocyte fusion to form syncytial myotubes and myofibers. Recently, researchers have also discovered a satellite-like cell, located on the surface of *Drosophila* IFMs, which requires Notch signaling for proliferation upon muscle injury (Chaturvedi et al., 2017).

Notch signaling has been shown to be upregulated with MeHg exposure in *Drosophila* C6 and C3 cell cultures, in *Drosophila* embryos, and in neuronal cell cultures (Bland and Rand, 2006; Rand et al., 2008; Tamm et al., 2008; Engel et al., 2012; Engel and Rand, 2014a). Curiously, in C6 cells, RNAi knockdown of either the Notch receptor or its co-activator Suppressor of Hairless had no effect on Enhancer of Split [E(spl)] upregulation by MeHg (Rand et al., 2008). This suggests that MeHg may induce transcription of E(spl)s in a receptor-independent mechanism of modulating Notch signals in the *Drosophila* model. *Drosophila* embryos exposed to MeHg also exhibited preferential upregulation of the Notch-target gene *Enhancer of split mdelta* [*E(spl)m*δ], in comparison to other genes known to respond to the activated Notch receptor (Engel et al., 2012), which also implies a Notch-independent mechanism. E(spl)mδ transcripts and an E(spl)mδ reporter gene exhibit expression in the myogenic domain in fly embryos, and E(spl)mδ has been suggested to play an essential role in mesodermal development (Wech et al., 1999; Engel and Rand, 2014a). Genetic upregulation of E(spl)mδ in the mesoderm of *Drosophila* embryos was also shown to cause a similar disruption of muscle formation as exposure to MeHg (Engel and Rand, 2014a). We therefore hypothesized that developing muscle is a direct target of MeHg and that E(spl)mδ is a muscle-specific mediator of MeHg toxicity.

In this study, we utilized the *Drosophila* model to examine the potential targeting of myogenesis by MeHg through genetic protection of muscle against MeHg exposure. As we have previously shown that Multidrug Resistance Protein (MRP) expression can modulate MeHg tolerance and susceptibility (Prince et al., 2014), we upregulated MRP in myogenic lineages and assessed MeHg tolerance through eclosion assays and IFM development. As E(spl)mδ upregulation has been implicated in MeHg exposure and is thought to be expressed in embryonic myoblasts and myotubes (Engel et al., 2012; Engel and Rand, 2014a), we furthermore assessed the role that E(spl)mδ plays in developing tissues as well as in mediating MeHg-induced myotoxicity. E(spl)mδ expression was modulated in a tissue-specific manner and in conjunction with MeHg exposure, and then the consequences on muscle morphology and eclosion behavior were assessed.

## Methods

### *Drosophila* stocks

The following *Drosophila* strains were obtained from the Bloomington *Drosophila* Stock Center (Indiana University, Bloomington, Indiana): Mef2-RFP (a recombinant of Mef2-GAL4 and UAS-RFP) (#26882); Mef2-GAL4 (#27390) (mesodermal driver); y^1^w^6723^ (YW) (#6599), Canton S. (CS) (#1); ELAV(1)-GAL4 (#458); MRP^EY11919^ [a Berkeley Drosophila Genome Project line that contains a 5′ promoter and upstream activation sequence (UAS)] (#20712) (Bellen et al., 2004); K33 (#6323); UAS-mδ RNAi/TM3, Sb (#26203), rebalanced with TM3,Ser,GFP; Attp2 control (#36303). UAS-mδ h8 was a gift from Sarah Bray (University of Cambridge, England) and is also available at Bloomington (#26677). NP1-GAL4 (gut epithelial driver) and Actin-GAL4/cyo,GFP (ubiquitous driver) were kindly provided by Benoit Biteau (University of Rochester, USA). The P3 E(spl)mδ deficiency line (Wurmbach and Preiss, 2014) was kindly provided by Annette Preiss (University of Hohenheim, Germany). P3 contains a deletion extending proximally from the K33 p-element and covers the entire E(spl)mδ gene; this deletion is homozygous lethal and therefore contains the TM6B balancer. UAS-E(spl)mδ ORF (#F000084), UAS-E(spl)mγ ORF (#F000131), and UAS-E(spl)m3 ORF (#F000090) lines (Bischof et al., 2013) were obtained from FlyORF.ch (University of Zurich, Switzerland). The UAS-SNS RNAi line (#109422) was obtained from Vienna Drosophila Resource Center (Vienna, Austria). The E(spl)mδ-GFP line was generated as described in Engel and Rand (2014a,b). Briefly, E(spl)mδ-GFP contains a 1.9 Kb region of the E(spl)mδ promoter upstream of GFP, which was cloned into the pGreen H-Pelican *Drosophila* transformation vector (Engel and Rand, 2014a). pGreen H-Pelican contains insulator sequences to avoid positional effects of chromosomal insertion. Flies were kept on a 12/12-h light/dark cycle in a 25°C humidified chamber on a standard fly food made of cornmeal, molasses, yeast, and agar.

### Eclosion assays

Tolerance of various *Drosophila* lines to MeHg was assayed by a previously described eclosion behavior assay (Mahapatra et al., 2010; Rand et al., 2014). A mating population of approximately 300 flies were prepared in small population cages equipped with an exchangeable grape-agar plate with a spot of yeast paste. Populations were composed of indicated crosses of virgin GAL4 females (approximately 200) with corresponding UAS males (approximately 100). Grape plates were exchanged after 8–14 h to collect successive embryo layings. Embryos were allowed to develop to first instar (L1) stage at 25°C. L1 larval offspring were then seeded at a density of 50 larvae per vial on vials of food (Jazz Mix, Fisher Scientific, #AS153) containing 0–20 μM MeHg (methylmercury chloride, Sigma-Aldrich # 215465), and allowed to develop for 13 days. Replicates of three vials for each MeHg concentration were achieved by collecting L1 larvae from separate embryo collection plates from the same mating population. After 13 days, flies that successfully eclosed (hatched from their pupal cases) were then counted.

Tissue-specific effects of overexpression of E(spl)mδ, E(spl)mγ, and E(spl)m3 on eclosion behavior were assayed in a similar manner as above using standard fly food without MeHg.

### Pupal staging and harvesting

Pupae were selected according to appearance of specific physical markers, as outlined in Bainbridge and Bownes (1981), since MeHg slows down *Drosophila* development, making time after pupal formation (APF) difficult to use (unpublished observations). Stages p5, p6, and p10 were chosen as they coincide with distinct phases of IFM development. Stage p5 occurs within 12.5–25 h of pupation (Bainbridge and Bownes, 1981), a time point at which myoblasts are migrating and fusing (Fernandes et al., 1991). Stage p6 occurs between 25 and 43 h of pupation (Bainbridge and Bownes, 1981), where myoblasts complete fusion and muscles elongate (Fernandes et al., 1991). Stage p10 occurs between 69 and 73 h of pupation (Bainbridge and Bownes, 1981), and coincides with muscle maturation. Pupae were determined to be at the p5 stage if they had undergone head eversion, and contained white Malpighian tubules within the interface of the thorax and abdomen. P6 pupae were selected upon the appearance of green Malpighian tubules within the abdomen, with a dark green “yellow body” at the anterior end of the Malpighian tubules, near the interface of the thorax and abdomen. P10 pupae selected upon the eyes turning a dark red color; orbital and ocellar bristles were present, but not thoracic bristles. Pupae were collected for either imaging or RT-qPCR, as described below.

### Imaging

Pupae were dissected out of their pupal case, at the indicated stages of development, and placed dorsally upward on double-stick tape on a glass slide. All pupae were imaged on a Nikon AZ100 Multizoom microscope (MVI, Avon MA). A total of 10–15 pupae were examined per treatment group. Muscle phenotypes were examined upon treatment with either 10 or 15 μM MeHg, concentrations, which are typically known to effect eclosion rates. No obvious phenotypes are seen with a 5 μM MeHg exposure. A range of phenotypes was found with MeHg treatment, making quantifications of phenotypes difficult. Therefore, quantification of MeHg effects was left to the eclosion assay, and representative images are shown here. A broader representation of the range of the phenotypes seen with MeHg exposure can be viewed in the Figures S1, S2.

### RT-qPCR

To examine MeHg effects on E(spl) gene expression, first instar larvae from a mating population of approximately 300 flies of the Canton S. strain were seeded at a density of 100 larvae/vial on fly food containing either 0 or 10 μM MeHg. For each of the 3 stages of development examined (p5, 6, and p10), pooled samples of 10 whole pupae were collected from 3 independent vials of either the 0 or 10 μM MeHg treatment groups. Replicates of three vials for each MeHg concentration were achieved by collecting L1 larvae from separate embryo collection plates from the same mating population. Pupae were homogenized in Trizol (Invitrogen) using a Kontes Pellet Pestle cordless motor (Fisher #NC0493674), and RNA from the indicated pupal stages was extracted using Trizol (Invitrogen). RT-qPCR was performed using the Biorad iScriptTM One-Step RT-PCR Kit with SYBR® Green kit (Biorad, # 170-8893), according to the manufacturer's protocol. Forty nanograms of RNA was used for each sample, and samples were run using a Biorad CFX Connect Real-time System. Fold change was calculated using the ΔΔCt method (Livak and Schmittgen, 2001). Samples were normalized to the ribosomal protein RP49 (aka, L32) reference gene to calculate ΔCt. RP49 was chosen as a reference gene as it is commonly used in qPCR gene expression analyses of *Drosophila* and other insect species (Daborn et al., 2002; Rand et al., 2008; Teng et al., 2012) and was also shown in this study to exhibit a variation within 0.5 Ct between all stages and treatments examined (Figure S3). The 10 μM treatment group was then normalized to the 0 μM control group to calculate ΔΔCt. Primer sequences were as follows, represented 5′/3′:

RP49: AGTATCTGATGCCCAACATCG/TTCCGACCAGGTTACAAGAAC
MRP: CTCAGTGGGCTAACGATCAAA/CAAATCCGAAGGCACCATAAAC
E(spl)mδ: CCGTTCAGGGTCAGAGATTTAT/CCTTGAGTTCGTCCAGATACAG (for determining mδ knockdown efficiency with Actin > mδ RNAi) and AGGATCTCATCGTGGACACC/CAGACTTCTTCGCCATGATG (for all other RT-qPCRs)
E(spl)mγ: GTCAATGAGGTCTCCCGTTC/GGTCAACAGGGAATGGCTGG
E(spl)m7: CGTTGCTCAGACTGGCGATG/ATCAGTGTGGTTCCAAAAGC
E(spl)mβ: GCTGGACTTGAAACCGCACC/AGAAGTGAGCAGCAGCCATC
E(spl)m3: AGCCCACCCACCTCAACCAG/CGTCTGCAGCTCAATTAGTC
Notch: GAATCTGCCCAGTCCGTAC/CCATTCATCCCGAGTCCT

### Statistical analysis

For RT-qPCR comparing 10 μM treatment to control treatment (0 μM), a two-tailed student-test was conducted. Fold change was calculated by comparing expression to that of the control treatment. A two-tailed student-test was also used for confirming upregulation or downregulation of Multidrug Resistance Protein (MRP) or E(spl)mδ; relative expression levels were expressed as a fold change, calculated by comparing expression to that of either the parent strain or the GAL4 crossed with the UAS parent strain, as indicated. All values are represented as an average of three replicates plus and minus standard error. *p* ≤ 0.05 were considered to be significant.

For RT-qPCR comparing expression levels of two independent UAS-mδ constructs to that of the Mef2-GAL4 parent line, a one-way ANOVA with Tukey's multiple comparisons test was conducted. All values are represented as an average of three replicates plus and minus standard error. *p* ≤ 0.05 were considered to be significant.

For eclosion assays, a two-tailed *z*-test was conducted, as the percent of flies successfully eclosed is a non-continuous value reaching 0 and 100% at the minima and maxima, respectively. Each MeHg concentration was treated categorically by comparing respective genetically manipulated strains or crosses to their relevant control strain or cross, as indicated. All values are represented as an average of three replicates plus and minus standard error. *p*-values of less than 0.05 or equal to were considered to be significant.

## Results

### Effects of genetic protection of muscle on MeHg tolerance and indirect flight muscle development

**Figure 1:**
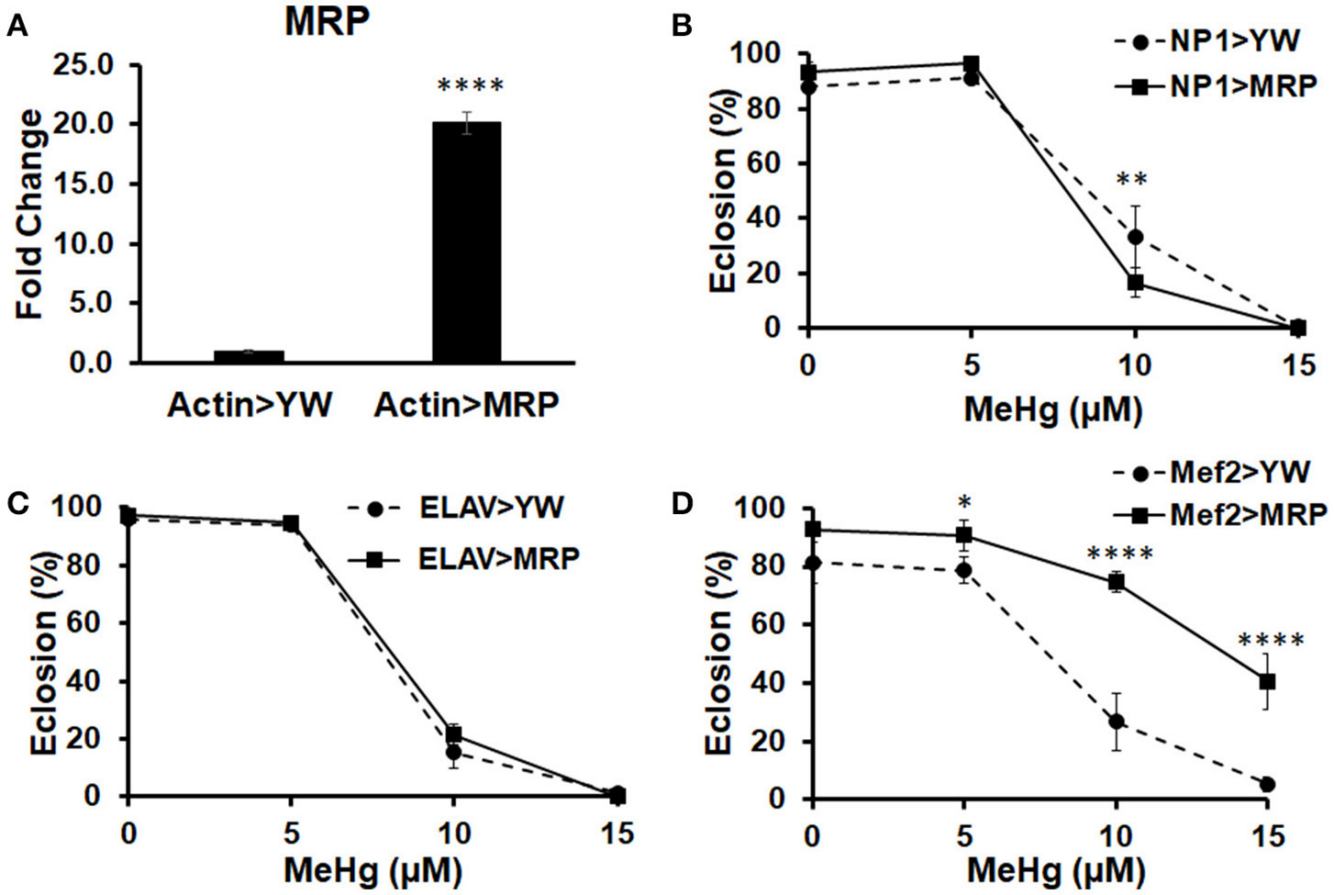
Multidrug Resistance Protein (MRP) upregulation in myogenic lineages conveys MeHg tolerance during development. **(A)** Expression of MRP, a xenobiotic and MeHg transporter, was assessed by crossing UAS-MRP (MRP^EY11919^) with Actin-GAL4 and conducting RT-qPCR on RNA extracted from pupal offspring (^****^p < 0.0001, *t*-test). Tolerance to MeHg during development was determined using an eclosion assay, with offspring of control (YW) or UAS-MRP flies crossed to various driver lines: **(B)** NP1-G4 (gut driver), **(C)** ELAV-G4 (neural driver), and **(D)** Mef2-G4 (muscle driver). Asterisks mark statistical significance in comparison to control at each treatment (^*^p ≤ 0.05, ^**^p ≤ 0.01, ^****^p < 0.0001, *z*-test).

We have previously shown that MeHg disrupts muscle development in *Drosophila*, both in embryos (Engel and Rand, 2014a) and in the indirect flight muscles (IFMs) during pupation (Montgomery et al., 2014). As neural and muscle development are highly dependent on each other (Fernandes and Keshishian, 1998; Landgraf et al., 1999), this finding could not distinguish whether or not MeHg-induced effects on muscle development may be a consequence of MeHg targeting of neurons. To test whether muscle is being directly affected, independent of effects that MeHg may have on neurons, we attempted to genetically protect muscle lineage cells by targeted upregulation of the Multidrug Resistance Protein (MRP) using the GAL4-UAS system (Figure 1A). MRP is a xenobiotic transporter that is known to excrete MeHg-glutathione complexes out of cells (Cernichiari et al., 2007). Tolerance to MeHg was assessed upon overexpression of MRP in muscle (Mef2-GAL4), neural (ELAV-GAL4), and gut (NP1-GAL4) tissues. We first evaluated eclosion behavior, which we have previously shown to be a sensitive read out of MeHg toxicity (Mahapatra et al., 2010; Rand et al., 2014). Eclosion is the very first behavior exhibited by the adult fly and requires a stereotypic peristaltic muscular contraction program known as extrication behavior (Reid et al., 1987). Eclosion rates are seen to decrease in a dose-dependent manner with developmental exposure to MeHg (Figure 1). Upon upregulation of MRP in gut tissue (NP1 > MRP), eclosion rates are slightly reduced at the 10 μM MeHg exposure in comparison to its control (NP1 > YW) (Figure 1B). Eclosion rates remain unchanged at all concentrations of MeHg when MRP is upregulated in neurons [ELAV(1) > MRP], in comparison to its control [ELAV(1) > YW] (Figure 1C). MRP upregulation in muscle (Mef2 > MRP), however, significantly increases the rate of eclosion on all concentrations of MeHg examined, in comparison to its control (Mef2 > YW) (Figure 1D).

**Figure 2:**
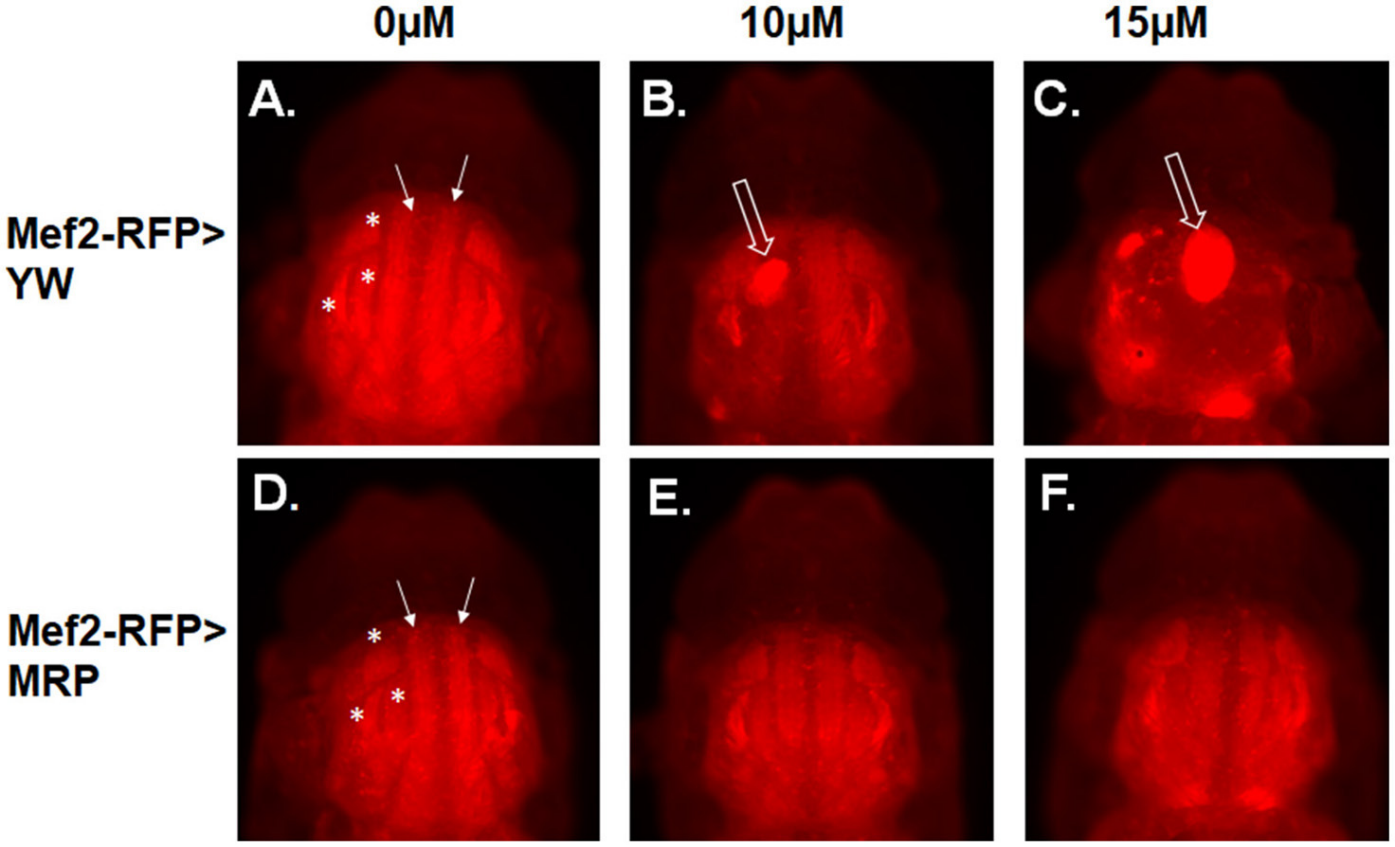
MeHg effects on indirect flight muscle (IFM) development are rescued with MRP upregulation. Epifluorescence images of IFMs of pupae at stage p10. **(A–C)** Mef2-RFP > YW (control) and **(D–F)** Mef2-RFP > MRP. Pupae were imaged after treatment with the indicated concentration of MeHg from the 1st instar larval stage. Asterisks mark the dorsal ventral muscles (DVM) and closed arrows mark the dorsal longitudinal muscles (DLM). Open arrows indicate failure of muscle fiber development (see Figure S1 for additional images).

To examine whether MRP upregulation could also rescue IFM perturbation by MeHg, we utilized the Mef2-RFP driver line to constitutively reveal muscle morphology via RFP expression while also driving MRP expression. Mef2-RFP > YW (control, Figures 2A–C) and Mef2-RFP > MRP (Figures 2D–F) 1st instar larvae were treated with MeHg concentrations known to inhibit eclosion, 10 and 15 μM MeHg, and allowed to develop to p10 pupae. Stage p10 was chosen as a representative culmination of muscle development. Normal flight muscle development is seen in the 0 μM treatment of both Mef2-RFP > YW (Figure 2A) and Mef2-RFP > MRP (Figure 2D) pupae, with the dorsal longitudinal muscles (DLM, closed arrows) and dorsal ventral muscles (DVM, asterisks) assuming a fully formed fiber morphology. With increasing concentrations of MeHg the IFMs of Mef2-RFP > YW are disrupted with muscles presenting in an aggregated mass (Figures 2B,C, open arrow). The flight muscles of Mef2-RFP > MRP are seen to be normal with exposure to 10 μM MeHg (Figure 2E), and only slightly disrupted at 15μM MeHg, but muscle fibers are clearly discernable (Figure 2F) (additional images in Figures S1A–C).

### Effects of developmental MeHg exposure on E(spl) expression in pupae

**Figure 3:**
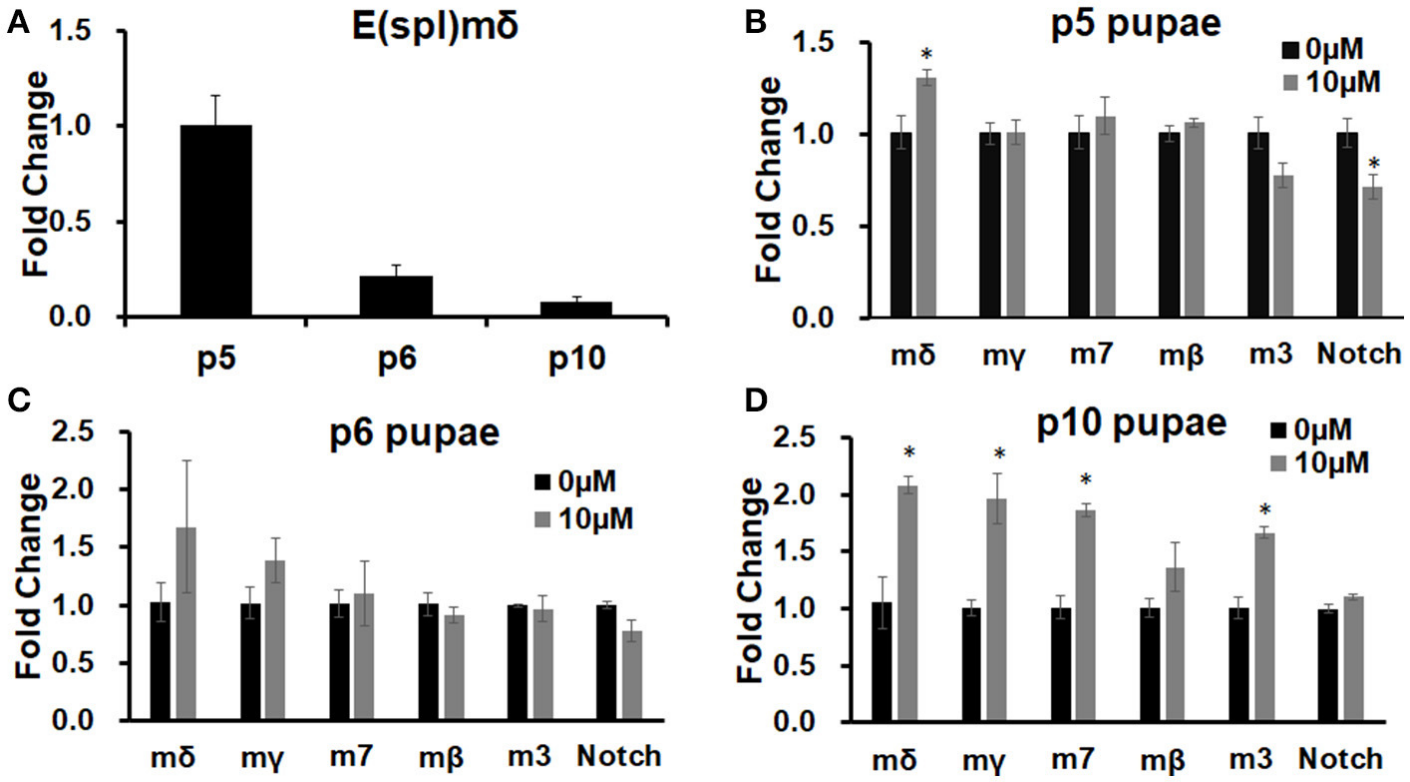
E(spl) expression in pupae developmentally exposed to MeHg. RT-qPCR on RNA extracted from pupae was performed to assess expression levels of E(spl)mδ and other E(spl) transcription factors. **(A)** E(spl)mδ expression over pupal development in Canton S. **(B–D)** Canton S. 1st instar larvae were exposed to either 0 or 10 μM MeHg and allowed to develop to **(B)** p5, **(C)** p6, and **(D)** p10 stages of pupal development. Asterisks mark statistical significance from respective 0 μM treatment (^*^p ≤ 0.05, *t*-test).

We have previously shown that *E(spl)m*δ is upregulated in embryos exposed to MeHg (Engel et al., 2012). We therefore examined if similar effects on E(spl) expression with MeHg exposure occurs across timepoints that represent distinct stages of IFM development in the pupae. The expression of *E(spl)m*δ RNA across normal pupal development is shown to be dynamic, with the highest expression at the p5 stage (12.5–25 h after pupal formation, APF; Figure 3A), a time when adult muscle precursor myoblasts (AMPs) are migrating and fusing with larval template muscles (Fernandes et al., 1991). *E(spl)m*δ expression shows a large decrease across the p6 (25–43 h APF) and into p10 (69–73 h APF) stages thereafter (Figure 3A). This is consistent with what has been reported for *E(spl)m*δ expression in the modENCODE temporal expression data set (Gelbart and Emmert, 2013). In comparison to control pupae, *E(spl)m*δ is significantly elevated with 10 μM MeHg exposure at the p5 stage (*p* = 0.036; Figure 3B). This upregulation occurs despite a slight decrease in *Notch* expression (*p* = 0.046; Figure 3B). Expression of other E(spl) genes is unmodified by MeHg exposure at this stage (Figure 3B). *E(spl)m*δ exhibits a trending increase, that does not reach significance, at stage p6 (Figure 3C), a time point where the majority of myoblasts are presumed to have fused with the IFM larval templates and muscle fibers begin extending to their tendon sites (Fernandes et al., 1991). The variation in the MeHg effect on *E(spl)m*δ levels seen at this stage likely stems from sampling within a developmental period where *E(spl)m*δ shows the greatest drop in endogenous expression (Figure 3A). Expression of *E(spl)m*δ, as well as *E(spl)m*γ*, E(spl)m7, and E(spl)m3* is significantly elevated, with respect to controls, with MeHg exposure at stage p10 (Figure 3D), a stage at which IFMs approach a mature fiber morphology. *Notch* and *E(spl)m*β expression is not seen to be altered with MeHg exposure at p10.

### E(spl)mδ expression in developing IFMs during pupation

**Figure 4:**
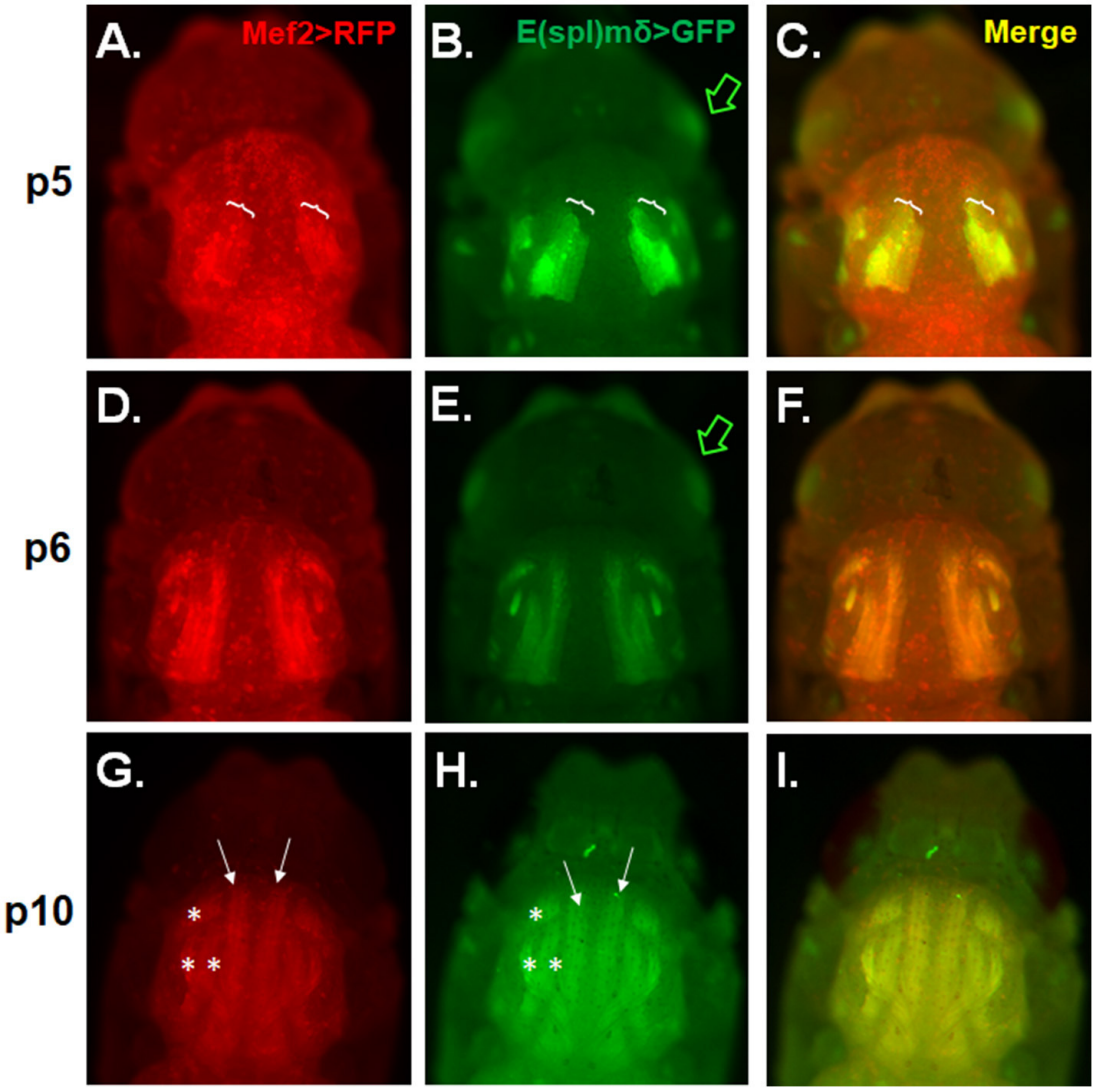
E(spl)mδ is expressed in the developing IFM. Live epifluorescent imaging of developing IFMs in pupae carrying both Mef2-RFP and E(spl)mδ-GFP at the indicated stages **(A–C)** p5, **(D–F)** p6, and **(G–I)** p10. **(C,F,I)** Merged images of RFP and GFP represent overlapping regions of expression of Mef2 in developing IFMs and E(spl)mδ. Pupae were dissected from their pupal cases and imaged directly. Asterisks mark the dorsal ventral muscles (DVM) and closed arrows mark the dorsal longitudinal muscles (DLM). Brackets mark the regions of DLM development that encompass myoblast fusing to the larval templates. Green arrow points to E(spl)mδ expression in the developing eye.

Because E(spl)mδ expression appears to localize to developing embryonic muscles (Engel and Rand, 2014a), we also wanted to see if E(spl)mδ is similarly expressed in developing adult flight muscles. IFM development was examined in pupae of various stages carrying a Mef2Gal4 > UASRFP and an E(spl)mδ enhancer-GFP reporter gene. RFP and GFP expression patterns were seen to largely overlap in the developing flight muscles in the thorax across the p5-p10 stages. At stage p5, Mef2-RFP and E(spl)mδ-GFP are seen to superimpose in the region where migrating myoblasts are presumably fusing with larval template (Figures 4A–C, brackets). E(spl)mδ-GFP is also seen to be expressed independently of Mef2 in the developing eye, a pattern that persists into p6 (Figures 4B,E green arrow). By stage p6, DLM fibers are clearly visible and expressing both RFP and GFP at a time where a majority of myoblasts have presumably fused with the larval templates and the growing fibers are extending out to their tendon sites of attachment (Figures 4D–F). By p10, the DLM (arrows) and DVM (asterisks) muscles are fully formed and visible with the perdurance of the RFP and GFP expression (Figures 4G–I). These data indicate the E(spl)mδ enhancer is activated in developing IFM muscle, suggesting that E(spl)mδ protein is likely expressed in this myogenic lineage.

### Effects of E(spl) overexpression in muscle, neurons, and gut

**Figure 5:**
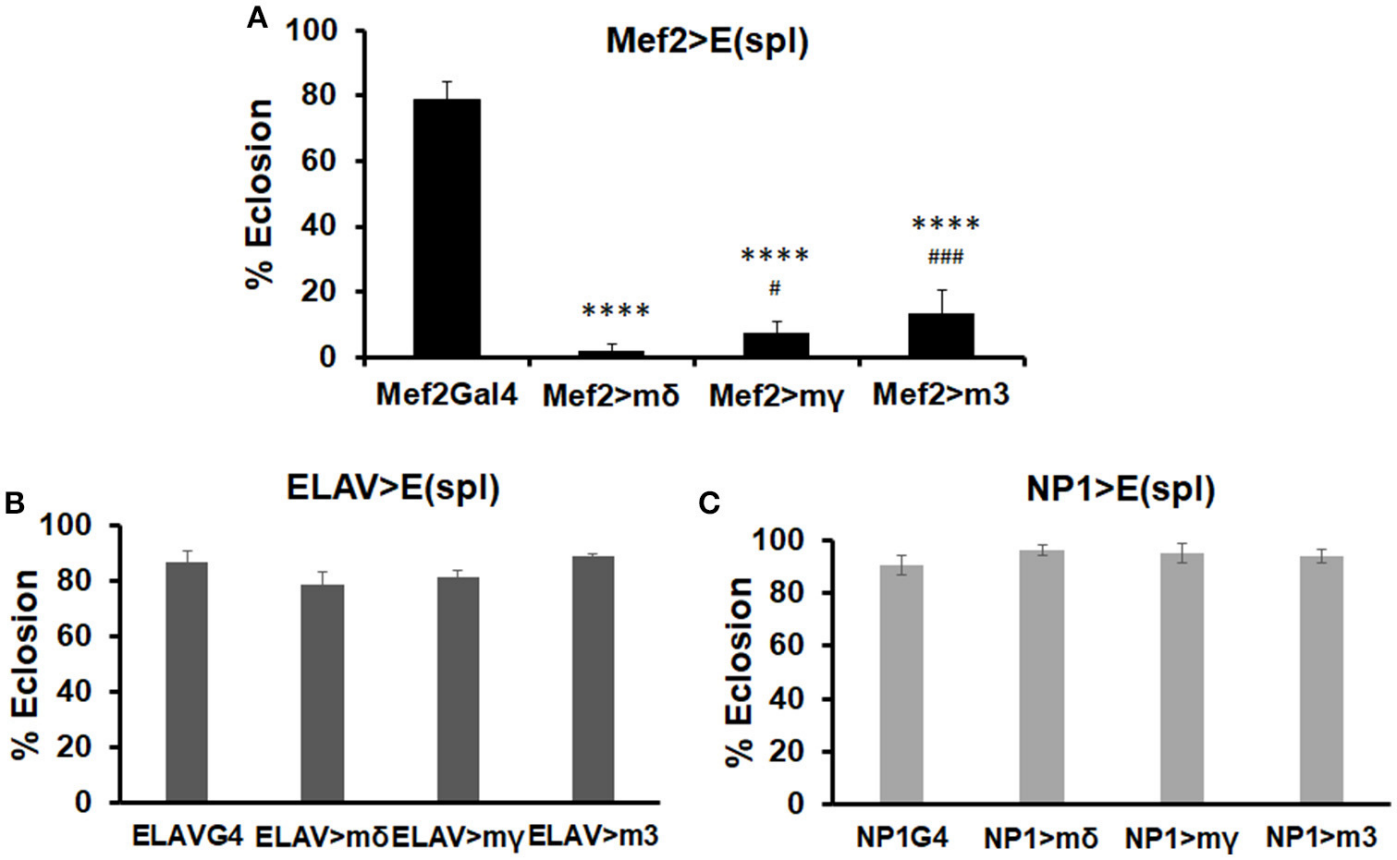
Effects on eclosion rate upon upregulation of various E(spl)s in neurons, gut, and muscle. Developmental effects of genetic upregulation of E(spl)mδ ORF, E(spl)mγ ORF, and E(spl)m3 ORF were assessed by eclosion assay in the absence of MeHg exposure. UAS-E(spl) ORF responders were crossed with various drivers: **(A)** Mef2-G4 (muscle driver), **(B)** ELAV-G4 (neural driver), and **(C)** NP1-G4 (gut driver). The number of flies successfully eclosed were scored (^****^p < 0.0001, in comparison to Mef2-Gal4; #*p* < 0.05, ###*p* < 0.001, in comparison to Mef2 > mδ, *z*-test).

To test whether E(spl)mδ upregulation by MeHg could directly affect muscle development and influence eclosion, we induced E(spl)mδ expression in the myogenic domain with the UAS-E(spl)mδ ORF responder (Figure 5A, Mef2-G4). For comparison, we also upregulated E(spl)mγ and E(spl)m3, by crossing Mef2-G4 with the corresponding UAS-ORF responders. The neuronal driver (ELAV-G4) and gut driver (NP1-G4) were also used for comparison of tissue-specific effects (Figures 5B,C). Upregulation of E(spl)mδ ORF in developing muscle almost completely inhibits eclosion (3% eclosion, Figure 5A). This effect is not specific to E(spl)mδ, as both E(spl)mγ ORF, and E(spl)m3 ORF greatly reduce eclosion rates (Figure 5B). However, both E(spl)mγ, and E(spl)m3 upregulation in muscle show slightly less of an effect on reducing eclosion rate (6 and 13%, respectively), compared to upregulation of E(spl)mδ (Figure 5A). In comparison, overexpression of these E(spl)s in neurons (Figure 5B) and gut tissue (Figure 5C) has no effect on eclosion rate.

### Flight muscle morphology and eclosion behavior upon upregulation of E(spl)mδ in myogenic lineages

**Figure 6:**
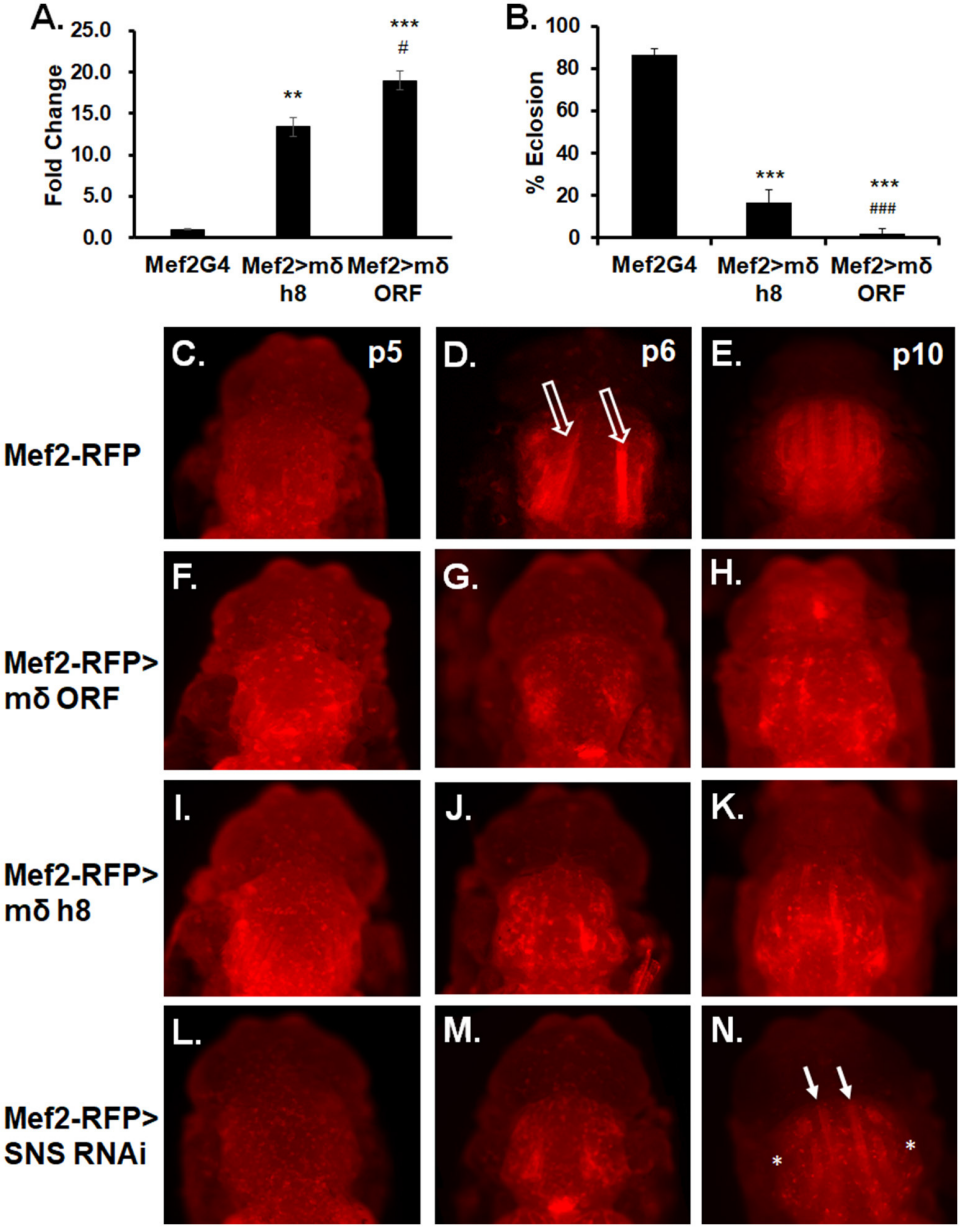
E(spl)mδ overexpression in myogenic lineage perturbs IFM development. **(A)** Expression levels of *E(spl)m*δ using two independent constructs, UAS-mδ h8 and UAS-mδ ORF, was assessed by RT-qPCR with RNA from p5 pupae (^**^p ≤ 0.01, ^***^p < 0.001 in comparison to Mef2-G4; #*p* ≤ 0.05, in comparison to Mef2 > mδ h8, one-way ANOVA)**. (B)** Eclosion of Mef2 > mδ h8 and Mef2 > mδ ORF (^***^p < 0.001 in comparison to Mef2-G4; ###*p* < 0.001 in comparison to Mef2 > mδ h8, *z*-test). **(C–N)** Developing IFMs in pupae at indicated stages were imaged by epifluorescence. Overexpression of E(spl)mδ was compared to knockdown of the myoblast fusion protein Sticks and Stones (SNS). **(C–E)** Mef2-RFP (control), **(F–H)** Mef2-RFP > mδ ORF, **(I–K)** Mef2-RFP > mδ h8, and **(L–N)** Mef2-RFP > SNS RNAi pupae were dissected from their pupal cases and imaged directly. Open arrows point to IFMs undergoing extension. White arrows point to partially formed DLM fibers. Asterisk mark absence of DVM formation.

Since MeHg causes only a moderate increase in *E(spl)m*δ expression levels, we next sought to determine the sensitivity effects of E(spl)mδ upregulation upon development and eclosion. Using a second responder line, UAS-mδ h8, which shows a 13-fold expression increase compared to a 19-fold increase seen with UAS-mδ ORF (Figure 6A), we assessed dose dependent effects of E(spl)mδ expression on eclosion rates. The increasing levels of E(spl)mδ expression seen with Mef2 > mδ h8 and Mef2 > mδ ORF, respectively, is seen to correspond with a decreasing eclosion rate (Figure 6B).

We next examined if the dose-dependent E(spl)mδ effect could be discerned in IFM morphology and what stage of muscle development might be sensitive to E(spl)mδ upregulation. For comparison, we also examined IFM development upon RNAi knockdown of Sticks and Stones (SNS), a core mediator of myoblast adhesion and fusion (Bour et al., 2000). Expression in the Mef2-RFP control reveals a normal pattern of flight muscle development across the p5-p10 stages (Figures 6C–E). In contrast, extending fibers are not visible in Mef2-RFP > E(spl)mδ ORF p6 pupae (Figure 6G), prossibly reflecting a failure in the preceding events of myoblast fusion to larval templates and/or larval template integrity. This pattern of muscle development failure with E(spl)mδ ORF expression persists into stage p10 (Figure 6H). Despite the lower E(spl)mδ expression level in Mef2-RFP > mδ h8 pupae (Figure 6A), the pattern and severity of the muscle development phenotype could not be discerned from that seen with Mef2-RFP > E(spl)mδ ORF (Figures 6I–K vs. Figures 6F–H).

With SNS RNAi, pupae at p6 stage show a similar pattern as seen with f Mef2-RFP > E(spl)mδ pupae (Figure 6M compared to Figures 6G,J). Muscle fibers are seen to form by p10 in Mef2RFP > SNS RNAi pupae (Figure 6N, green arrows); however, DLM fibers appear much thinner than normal, and some DVMs are not apparent (Figure 6N, asterisks).

### Effects of E(spl)mδ knockdown on MeHg tolerance and IFM development

**Figure 7:**
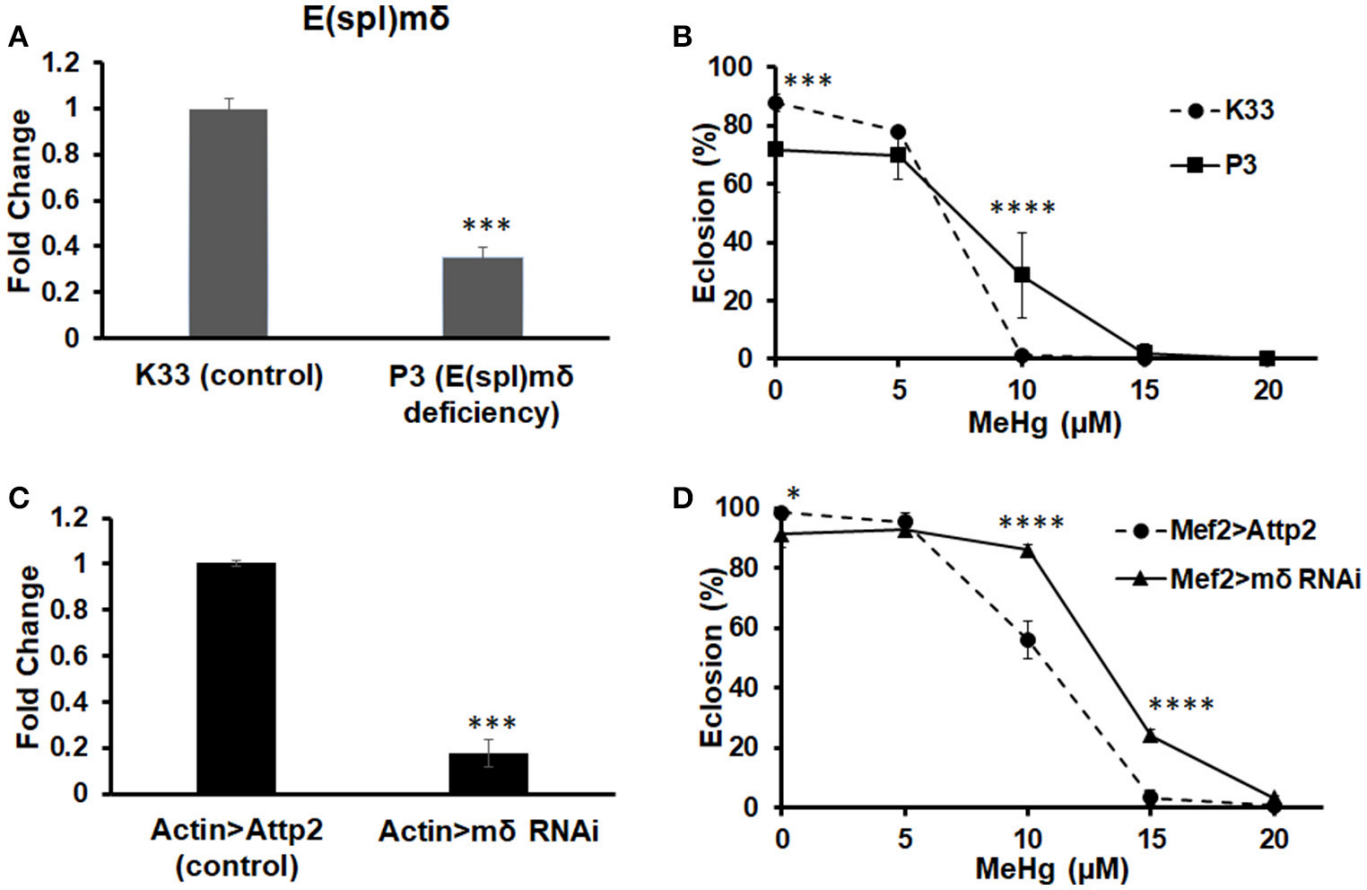
Knockdown of E(spl)mδ conveys MeHg tolerance during development. Expression of *E(spl)m*δ was assessed by RT-qPCR with RNA extracted from p5 pupae of **(A)** E(spl)mδ deficiency (P3) and its background control strain (K33) and **(C)** Mef2 > E(spl)mδ RNAi and Mef2 > Attp2 (control) (^***^p < 0.001, *t*-test). Tolerance to MeHg was determined through an eclosion assay of **(B)** E(spl)mδ deficiency (P3) and its background control strain (K33) and **(D)** Mef2 > E(spl)mδ RNAi and Mef2 > Attp2 (control) strain (^*^p ≤ 0.05, ^***^p < 0.001, ^****^p < 0.0001, *z*-test).

To examine if E(spl)mδ is a muscle-specific mediator of MeHg toxicity, we tested the effects of reducing *E(spl)m*δ expression in conjunction with MeHg exposure on eclosion and IFM development. Tolerance to MeHg upon reduction of *E(spl)m*δ expression was examined using an E(spl)mδ deficiency line (P3) as well as an RNAi against *E(spl)m*δ. The P3 E(spl)mδ deficiency line, which was necessarily maintained as a heterozygote, exhibits an *E(spl)m*δ expression level at 35% of the K33 control strain (Figure 7A). RNAi knockdown with the ubiquitous actin driver (Actin-G4) results in *E(spl)m*δ expression at 17% of control levels (Figure 7C). The P3 E(spl)mδ deficiency shows a greater tolerance to MeHg relative to its control strain (K33), as seen by an increase in eclosion rate at the 10 μM MeHg treatment level (Figure 7B). In comparison, RNAi knockdown of E(spl)mδ in the muscle domain, exhibits an even greater tolerance to MeHg, with increased eclosion rates at 10 and 15 μM MeHg exposures (Figure 7D). The greater tolerance to MeHg of both the P3 strain and the Mef2 > mδ RNAi cross is despite small but significant decreases in eclosion rates at the 0 μM MeHg treatment (Figures 7B,D).

**Figure 8:**
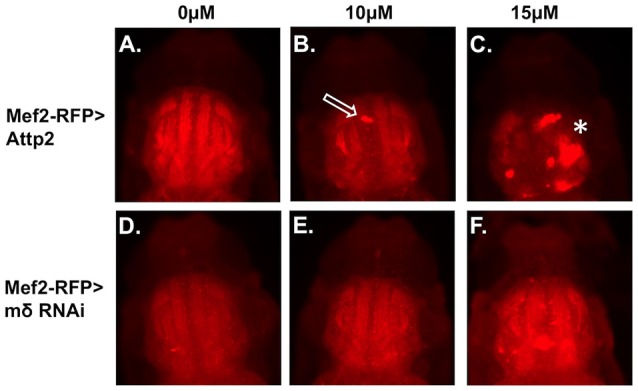
Rescue of MeHg effects on IFM development with downregulation of E(spl)mδ in myogenic lineages. Developing IFMs in pupae, at the p10 stage after treatment with the indicated concentrations of MeHg were imaged by epifluorescence of **(A–C)** Mef2-RFP > Attp2 (control) and **(D–F)** Mef2-RFP > mδ RNAi. Pupae were dissected from their pupal cases and imaged directly. Asterisks and arrows mark failure of IFM development (see Figure S2 for additional images).

Effects of E(spl)mδ knockdown on muscle morphology were also examined in p10 pupae exposed to 0, 10, and15 μM MeHg (Figure 8). At 10 μM MeHg, the Mef2-RFP > Attp2 control exhibits evidence of some IFM defects (Figure 8B, open arrow); whereas at 15 μM, the muscle fibers of Mef2-RFP > Attp2 are non-existent. Aggregates of RFP expressing bodies in the thorax suggest that myoblast fusion may have occurred but that DLM and DVM fiber elongation has failed (Figure 8C, asterisk). In comparison, muscle fiber development, is largely rescued from the effects of 10 and15 μM MeHg treatment with expression of E(spl)mδ RNAi in muscle (Mef2-RFP > mδ RNAi) (Figures 8E,F, asterisk and open arrow) (Additional images in Figures S2A–C).

## Discussion

We have shown here that protecting developing muscle from the effects of MeHg, through the upregulation of MRP in the muscle domain, can rescue the overall development of the fly. This can be seen both in an increase in the flies' ability to eclose and in a rescue of the effects of MeHg on adult flight muscle morphology. Using the same strategy to protect neuronal or gut tissue did not show an increased tolerance to MeHg, as measured by eclosion rates. These data suggest that muscle development can be targeted by MeHg through a mechanism independent of the effects of MeHg on neural development. Although the dose levels used in this study, namely 5–20 μM MeHg in the fly food are high in comparison to typical human exposures [e.g., <0.1–1 ppm (<0.5–5 uM) MeHg in dietary fish], MeHg exposure levels used here are gauged with respect to observable effects elicited in this model system in order to gain insights into mechanism.

One mechanism by which MeHg may be disrupting muscle development is through modulation of the tissue-specific effectors in the Notch signaling pathway. The tissue specificity of transcriptional factors downstream of Notch remains an active area of research. Several studies have suggested that the function of the mammalian Hes/Hey and *Drosophila* E(spl) family of proteins are redundant (Fischer et al., 2004; Macdonald et al., 2005; Buas et al., 2010; Wurmbach and Preiss, 2014). However, the expression patterns of these proteins are distinct and may act in a timing and tissue-dependent manner, depending on tissue-specific regulators (de Celis et al., 1996; Wech et al., 1999). In *Drosophila*, Twist is thought to be a co-regulator of Notch in myogenesis, conferring a specific response to Notch signaling (Bernard et al., 2010). In vertebrates, overexpression of the Notch transcriptional repressors Hes6 and Hey1 have been shown to inhibit myogenic differentiation, while HeyL overexpression has little effect on differentiation (Cossins et al., 2002; Buas et al., 2009, 2010). Additionally, prior studies in *Drosophila* embryos, using transcript hybridization and a GFP reporter for a 1.9 kb E(spl)mδ enhancer, have suggested that E(spl)mδ is expressed in the myogenic domain (Wech et al., 1999; Engel and Rand, 2014a). Here, we demonstrate, using the same GFP reporter, that E(spl)mδ expression is likely localized to the myogenic lineage giving rise to the adult IFM. Furthermore, overexpression of E(spl)mδ in developing muscle, in contrast to a similar overexpression in neurons or gut tissue, can disrupt development and reduce eclosion rates. While it cannot be concluded from these results that E(spl)mδ plays an endogenous role in IFM development, our data suggest that in comparison to other tissues, E(spl)mδ may be a target of MeHg toxicity in developing muscle.

In response to MeHg, *E(spl)m*δ appears to be upregulated in *Drosophila* pupae across various stages of IFM development. It remains uncertain as to the mechanism of *E(spl)m*δ upregulation. Although *E(spl)m*δ is known to respond to activation of the Notch receptor (Wurmbach et al., 1999), MeHg may be upregulating *E(spl)m*δ in a Notch-independent manner. Previous data has shown that various E(spl) repressors are upregulated in *Drosophila* C6 cells in response to MeHg through a Notch-independent mechanism (Rand et al., 2008). A similar Notch-independent mechanism of E(spl) activation *in vivo* remains to be demonstrated. However, in both *Drosophila* embryos and in *Drosophila* pupae, shown here, MeHg somewhat preferentially acts upon *E(spl)m*δ relative to other E(spl) genes (Engel et al., 2012). This preferential upregulation of a single *E(spl)* gene may also be indicative of an novel Notch-independent mechanism of MeHg activity. However, this mechanism will require further study both *in vitro* and *in vivo*.

Here, we examined gross morphogenic phenotypes in adult flight muscle (IFMs) to ascertain which global events and stages of muscle development might be affected by MeHg toxicity and point to possible underlying mechanisms. For example, strong upregulation of Notch signaling, via activated Notch expression, has previously been shown to cause persistent Twist expression in the AMPs of developing IFMs, leading to a complete loss of the IFMs (Anant et al., 1998). It is possible that a more restricted activation of the Notch target E(spl)mδ via MeHg may suppress AMP differentiation more moderately, giving rise to the phenotypes seen here. Notch signaling also modulates myoblast adhesion and fusion events in the progression of IFM formation, and has been shown to influence expression of the canonical myoblast fusion proteins SNS and Kirre (Gildor et al., 2012). Here, we find that altering SNS expression yields disrupted patterns of IFM development that partially mimic effects seen with MeHg. Remarkably, *Sns* and *Kirre* have been associated with MeHg tolerance and susceptibility through a genome wide association study in *Drosophila* (Montgomery et al., 2014). The possibility that E(spl)mδ may influence SNS or Kirre expression therefore also remains an attractive hypothesis to explore.

Despite several similarities, MeHg treatment and E(spl)mδ upregulation exhibit some phenotypic differences. Unlike in genetic E(spl)mδ upregulation, it appears myoblast fusion events do occur with MeHg treatment, as suggested by the large aggregates of Mef2 expressing bodies in the thorax. These differences may reflect the level of E(spl)mδ expression that is achieved with each approach, since eclosion rates were seen to be sensitive to E(spl)mδ dose. Alternatively, these differences may also be caused by MeHg targeting of additional factors involved in muscle development. Genes involved in neuromuscular junction formation and attachment to tendon cells have also been implicated in MeHg susceptibility (Montgomery et al., 2014). More research will be needed to elucidate these mechanisms at the level of cell-cell interactions.

Nonetheless, our data support the conclusion that E(spl)mδ is a mediator of MeHg toxicity in *Drosophila* muscle development. Furthermore, E(spl)mδ activity demonstrates tissue-specificity in that developing muscle appears to not only express E(spl)mδ, but is especially sensitive to genetic modulation of this transcription factor. Overall, these data elucidate an important mechanism by which modulation of the Notch target gene E(spl)mδ by the environmental toxicant MeHg can have tissue-specific implications. Establishing muscle development as a direct target of MeHg toxicity will bring greater understanding of the etiology of motor deficits typically seen with elevated environmental exposure to MeHg.

## Author contributions

Conceived and designed the experiments: MR and LP. Performed the experiments: LP. Analyzed and interpreted the data: MR and LP. Drafted the paper: LP. Revised the paper for intellectual content: MR.

### Conflict of interest statement

The authors declare that the research was conducted in the absence of any commercial or financial relationships that could be construed as a potential conflict of interest.
