# Supplementary material for: Notch Target Gene E(spl)mδ Is a Mediator of Methylmercury-Induced Myotoxicity in Drosophila
Source: Front Genet. 2018 Jan 15;8:233. doi: 10.3389/fgene.2017.00233 (PMC5775289; doi:10.3389/fgene.2017.00233)
Supplement: Supplementary file 1 [file Image1.pdf]

Suppl Figure 1A

Mef2-RFP>YW 0μM MeHg

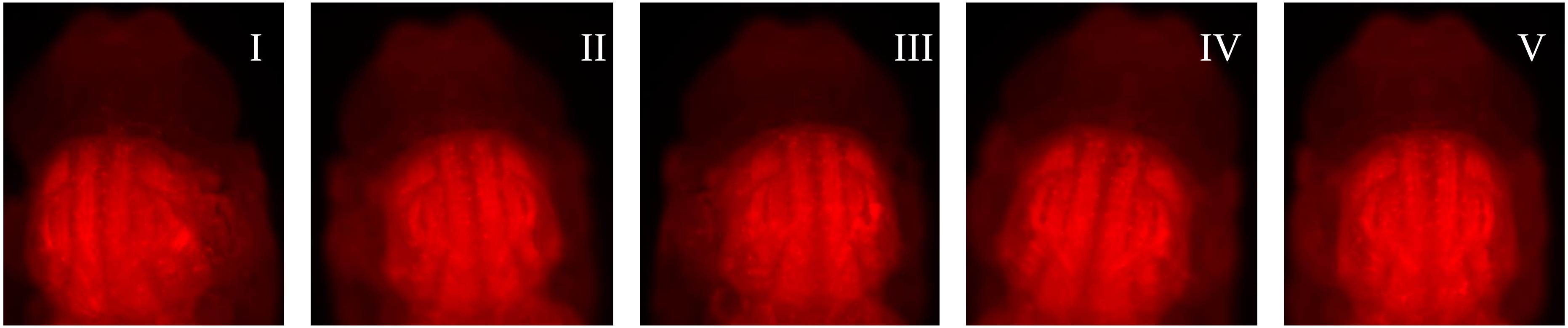

Mef2-RFP>MRP 0μM MeHg

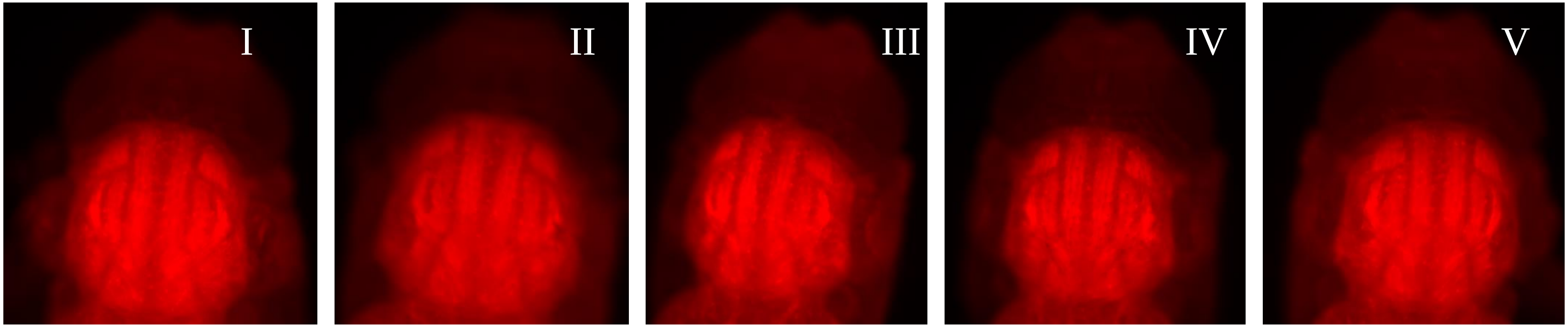

Suppl Figure 1B

Mef2-RFP>YW 10μM MeHg

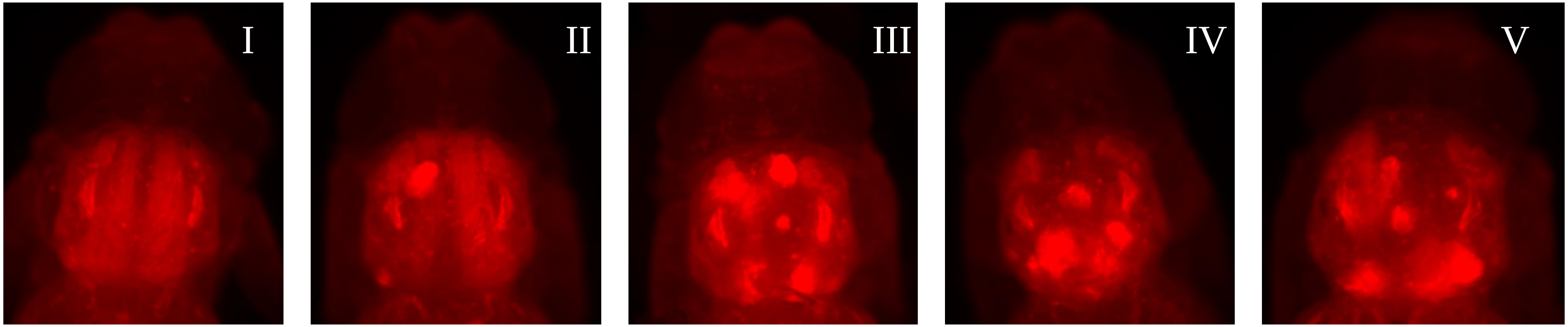

Mef2-RFP>MRP 10μM MeHg

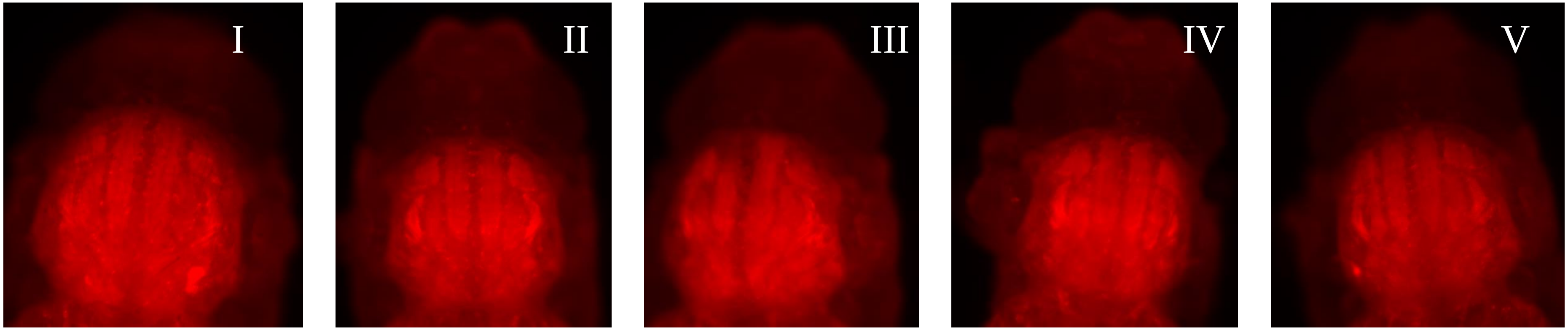

Suppl Figure 1C

Mef2-RFP>YW 15µM MeHg

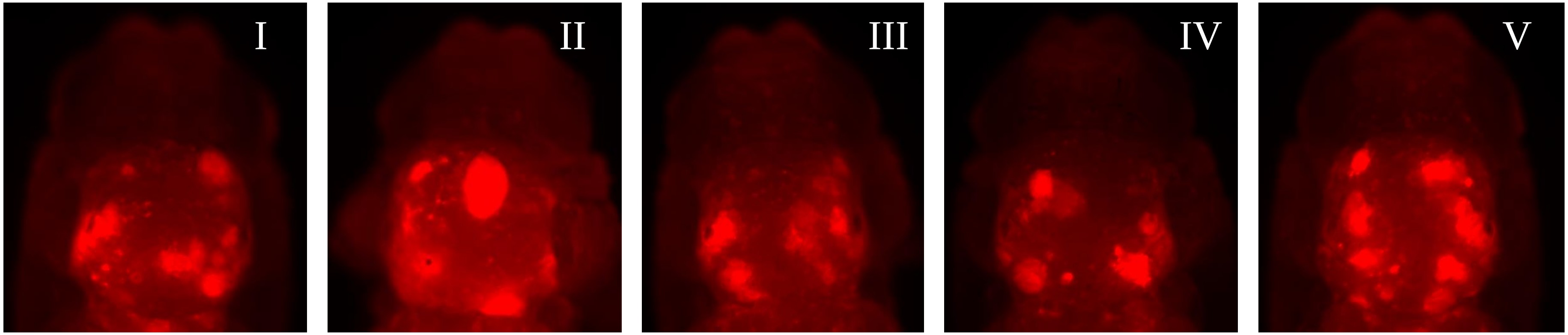

Mef2-RFP>MRP 15µM MeHg

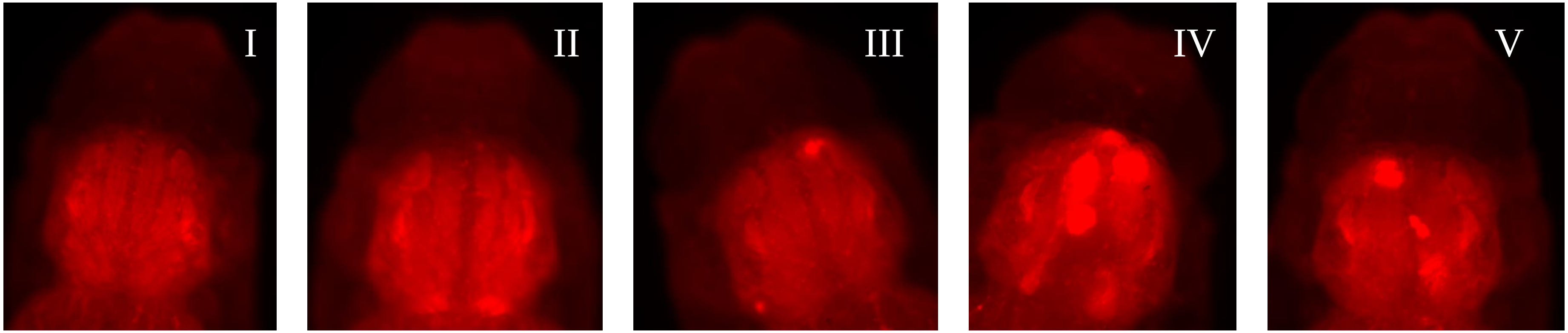

Fig S1. Range of phenotypes upon MeHg exposure in Mef2>MRP and Mef2>YW pupae. Epifluorescence images of IFMs of pupae at stage p10, exposed to A.) 0µM, B.) 10µM, and C.) 15µM MeHg. (Images in 1B II and 1C II are also shown in Fig. 2, B,E, and C,F, respectively)

Suppl Figure 2A

Mef2-RFP>Attp2 0μM MeHg

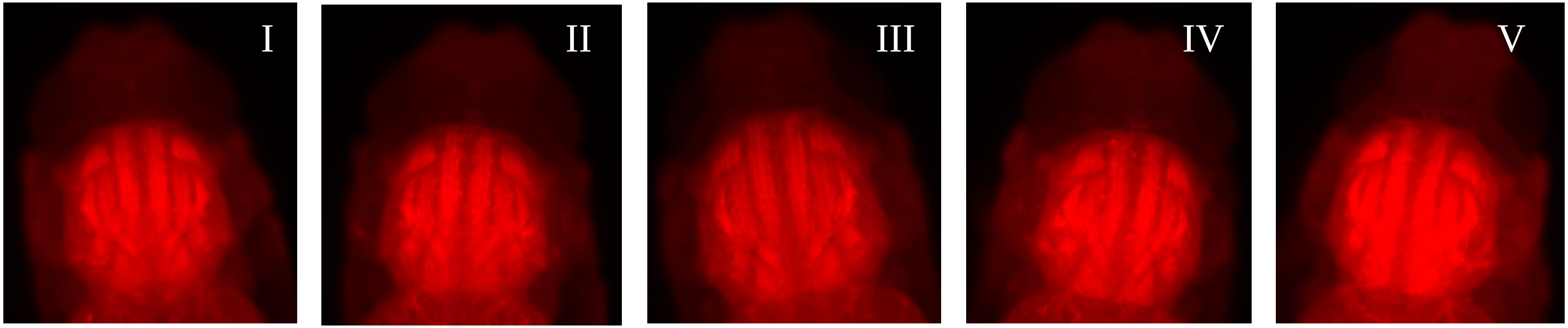

Mef2-RFP>E(spl)mδ RNAi 0μM MeHg

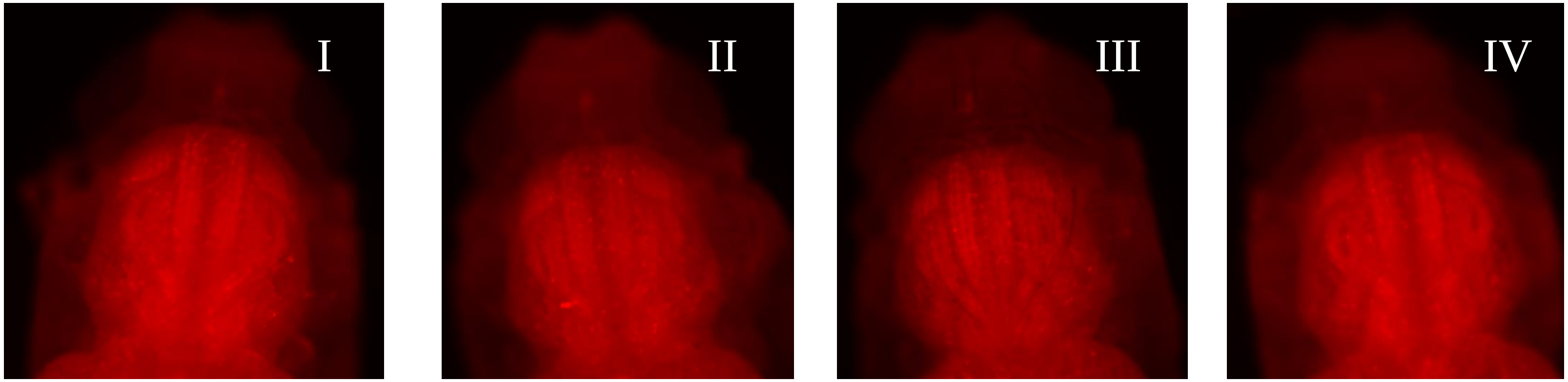

Suppl Figure 2B

Mef2-RFP>Attp2 10μM MeHg

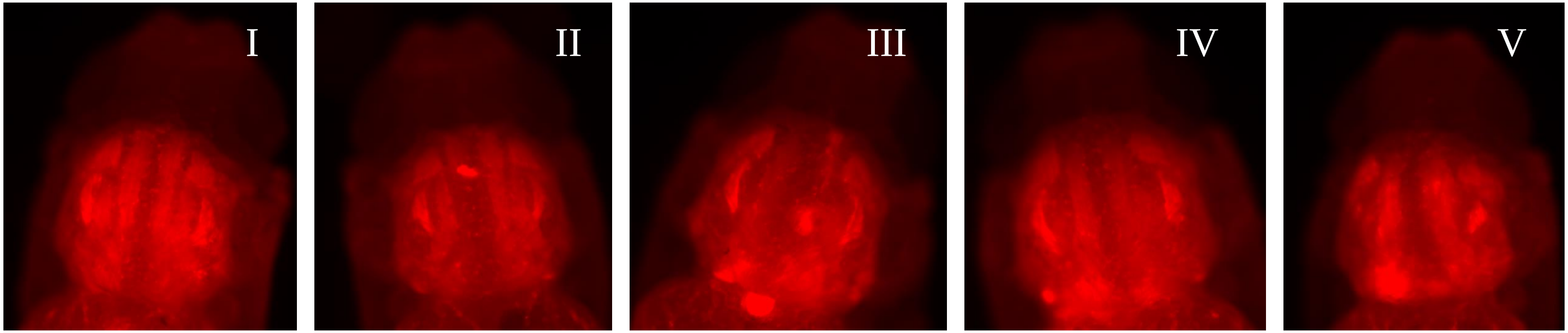

Mef2-RFP> E(spl)mδ RNAi 10μM MeHg

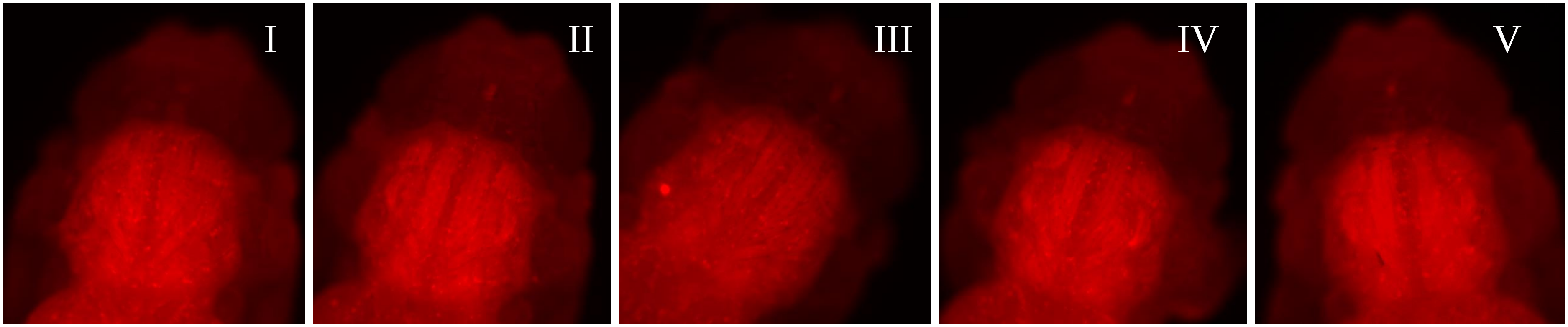

Suppl Figure 2 C

Mef2-RFP>Attp2 15µM MeHg

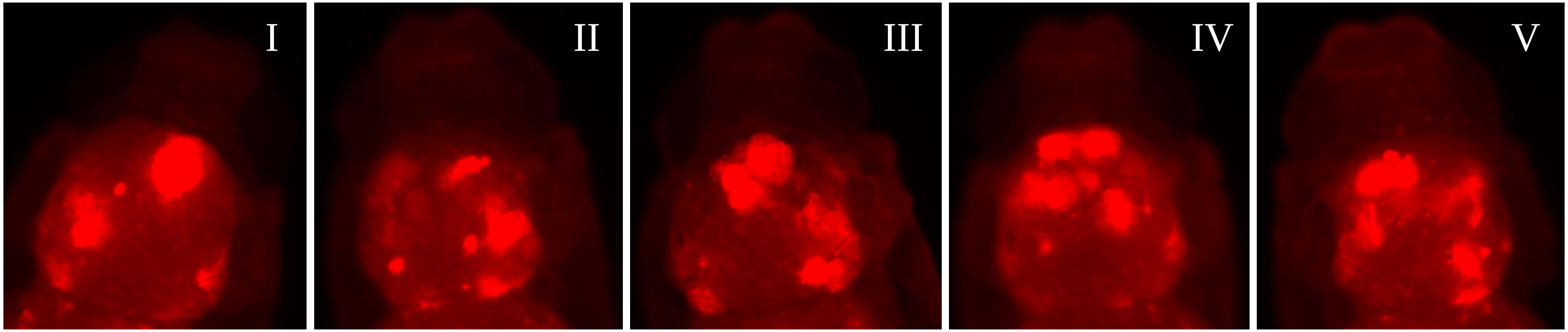

Mef2-RFP>E(spl)mδ RNAi 15µM MeHg

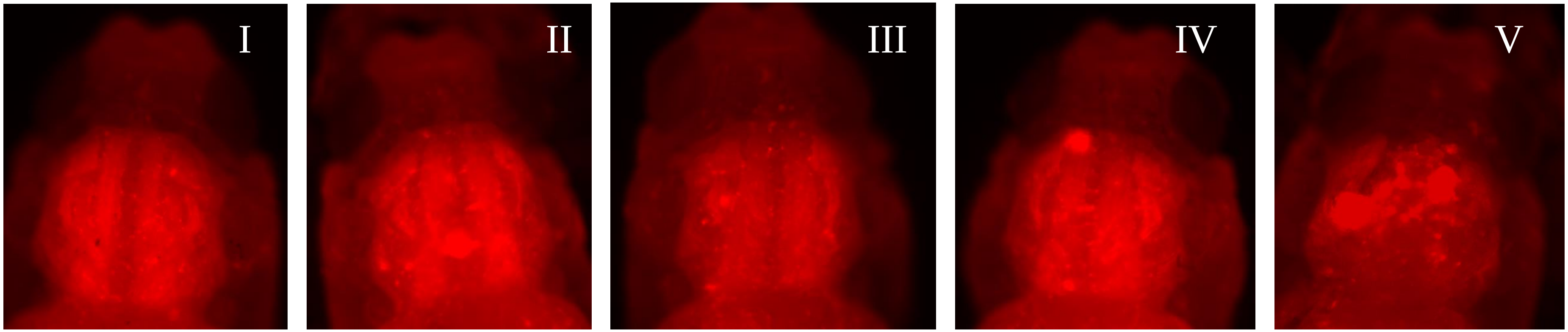

Fig S2. Range of phenotypes upon MeHg exposure in Mef2>mδ RNAi and Mef2>Attp2 pupae. Epifluorescence images of IFMs of pupae at stage p10, exposed to A.) 0µM, B.) 10µM, and C.) 15µM MeHg. (Images in 2A II, 2B II and 2C II are also shown in Fig. 8, A,D, 8 B,E, and 8 C,F, respectively)

Suppl Figure 3

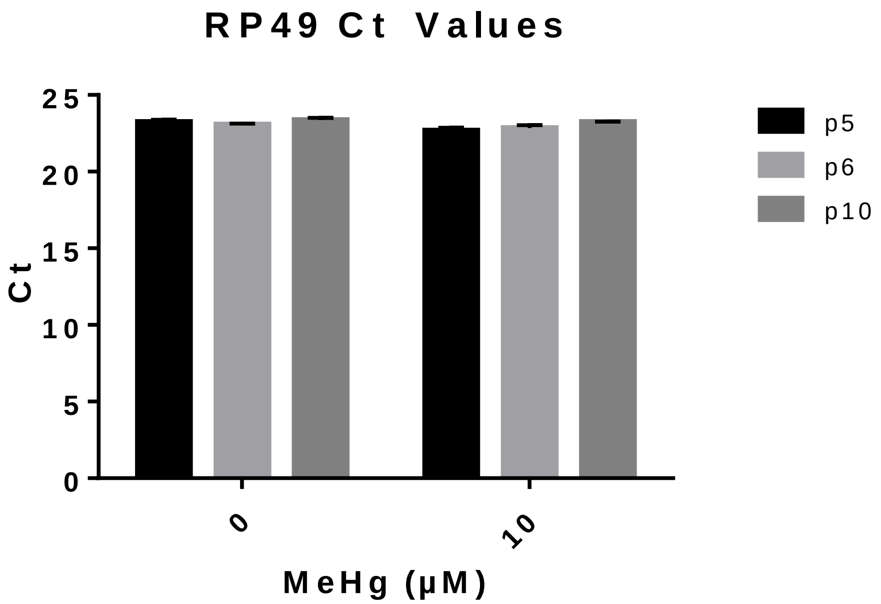

Fig S3. RP49 Ct values. RP49 Ct values of Canton S pupae at stages p5, p6, and p10. Pupae were exposed to either 0µM or 10µM from 1st instar larval stages.
